# ‘Read for Nutrition’ programme improves preschool children’s liking and consumption of target vegetable

**DOI:** 10.1017/S1368980021004985

**Published:** 2022-05

**Authors:** Maha Elrakaiby, Saima Hasnin, Virginia C Stage, Dipti A Dev

**Affiliations:** 1 Department of Child, Youth and Family Studies, University of Nebraska-Lincoln, Lincoln 68588-0364, USA; 2 Department of Nutrition Science, College of Allied Health Sciences, East Carolina University, Greenville, USA

**Keywords:** Preschool children, Vegetable consumption, Vegetable liking, Childcare, Programme evaluation

## Abstract

**Objective:**

To determine whether the ‘Read for Nutrition’ programme would increase liking and consumption of broccoli (a target vegetable) in preschool children and test acceptability and practicality of the programme.

**Design:**

Pilot pre-post intervention study, where childcare teachers received training and coaching followed by reading the book ‘Monsters Don’t Eat Broccoli’ multiple times with the children during a three-week intervention.

**Setting:**

Five classrooms of Educare, Lincoln, Nebraska in 2018.

**Participants:**

Sixty-nine (11 to 16 children per classroom) preschool-aged children and sixteen teachers (minimum, three per classroom).

**Results:**

Average total consumption of broccoli increased 35 % (0·14 ounces or 0·05th cup) after the ‘Read for Nutrition’ programme (*t* = 2·66; *P* = 0·01; 95 % CIs (0·035, 0·246)) for all children. Proportional consumption increased for children who received ≥ five exposures to the book (*t*_*46*_ = 2·77; *P* = 0·008). Exposures to the book predicted proportional consumption (*β* = 0·365; *P* = 0·002). Liking of broccoli increased (*W*_*69*_ = 2·2, *P* = 0·03) as well. Teachers rated the programme as acceptable, practical and enjoyable to children and to themselves.

**Conclusions:**

Programmes such as ‘Read for Nutrition’ have the potential to improve children’s vegetable liking and consumption in early care and education settings with only book readings and no exposure to a real vegetable.

Eating fewer vegetables in early childhood may increase children’s risk of obesity and associated chronic diseases in adulthood^(1–3)^. Moreover, vegetables provide important nutrients necessary for optimal growth and development in preschool children^(3)^. Yet, nine in ten children do not eat the minimum recommended amount of vegetables^(4)^. Therefore, calls for action have been made to improve children’s vegetable liking and consumption as an avenue for preventing childhood obesity^(5,6)^. Since research has shown that eating behaviours developed in childhood track into adolescence and adulthood, preschool age is a critical developmental period to shape children’s vegetable liking^(7,8)^.

Early care and education (ECE) settings offer an ideal environment to improve children’s vegetable consumption^(9,10)^. Preschool children consume at least half to three-quarters of their daily dietary intake in the ECE settings when enrolled full-time^(11)^. To increase exposure and consumption of vegetables among preschool children the Child and Adult Care Food Programme policies require the participating ECE setting serve vegetables during meals and encourage serving vegetables during snacks^(12)^. However, teachers have reported challenges regarding encouraging preschool children to eat vegetables^(13)^ and have shared concerns about children’s food refusal when vegetables are served^(14)^. Additionally, both teachers’ and children’s taste preferences, children’s food neophobia and picky eating have been reported as other challenges to improving vegetable consumption in preschool children^(2,15)^.

To encourage children to eat more vegetables, evidence-based practices (EBP) have been implemented during and outside of the mealtime setting. EBP for enhancing children’s vegetable consumption during mealtime include enthusiastic role modelling^(16)^, engaging children’s senses^(17)^ and verbal praise^(18)^. When adult caregivers talk positively and enthusiastically about a vegetable and express their liking of it, children may be more likely to try the vegetable^(16)^. Similarly, when children are asked questions about the colour, smell, sound, texture or taste of the vegetable, they may be more willing to try it^(17)^. Praising children for trying a vegetable may also increase their vegetable consumption^(18)^. However, nutrition education during mealtimes is sub-optimal, and teachers have reported they are pre-occupied with other mealtime duties and children are distracted^(19–21)^. Therefore, improving vegetable consumption in children during daily ECE routines offers an alternative and promising approach. The alternative approaches that can be implemented during daily ECE routines include repeated exposure (8–15 times) to the target vegetable, non-taste sensory education, learning about the health benefits of foods and interactive shared book reading with sensory exploration^(22)^.

Although repeated exposure may improve children’s target vegetable consumption, caregivers abandon efforts as they believe that the child will not eat the food after 3-to-5 exposures^(23)^. Additionally, ECE teachers have reported limited financial resources to buy fresh foods for hands-on nutrition education rather than for consumption^(24)^. Consequently, books offer a feasible alternative for increasing children’s exposure to vegetables in the ECE settings^(25,26)^. Previous research has demonstrated that repeatedly looking at pictures of vegetables in books, reading books with vegetable and character congruence (e.g. carrots and rabbit), interactive shared book reading and actively engaging children can enhance children’s visual liking and willingness to taste the featured vegetables^(25–27)^.

Given the teacher-reported challenges to encourage children to consume vegetables, it is important to determine the impact, acceptability and practicality of an ECE professional development book reading programme. The ‘Read for Nutrition’ programme was designed to (a) expose the children to a bitter-tasting target vegetable (broccoli) through reading a congruent book instead of using real food tasting activities; (b) integrate and translate the EBP with the goal of empowering teachers to implement these strategies during book reading routines with preschool children in the natural ECE setting and (c) determine the acceptability and practicality of implementing combinations of EBP (role modeling, engaging children’s senses and repeated exposures without using real foods) in a natural ECE non-mealtime setting. We chose broccoli as the target vegetable as it is commonly referred as bitter tasting and children may have a natural tendency to dislike bitter tasting vegetables^(28,29)^. Moreover, it has been known as a familiar vegetable in this age group^(30,31)^ and can be served raw with minimal preparation.

It was hypothesised that the programme will increase children’s consumption and liking of the target vegetable and that the programme would be acceptable and practical in a natural ECE setting.

## Materials and methods

### Study design

The current study is a pre-post design pilot study for evaluation of the ‘Read for Nutrition’ programme. The sample size was determined using G × Power 3·1 where alpha was set to 0·05, power to 0·8 and effect size to 0·5 (medium effect size)^(32)^. A sample of 45 children was needed. Data were collected before (pre-test) and after the intervention was completed (post-test) (3 weeks). The study was conducted January–May 2018 and was approved by University of Nebraska-Lincoln Institutional Review Board.

### Participants and recruitment

A large, single childcare site was selected rather than multiple sites with fewer children to avoid extraneous influences that would be difficult to control in data analyses when measuring vegetable consumption. Such factors include diverse types of preparation and varieties of vegetables served at ECE meals; teachers’ practices; meal service; nutrition-education resources; and centre-level policies regarding mealtime practices for children and teachers. Furthermore, Educare was conveniently selected because the childcare centre caters to children ages 3–5 years from low-income families, is licensed by the Nebraska state regulatory agency, participates in the Child and Adult Care Food Programme, and majority of the children attending Educare are dual language learners^(33)^. Lastly, the centre has previously collaborated in research projects with the authors.

Primary participants for the current study were 3–5-year-old children from five classrooms along with ECE teachers that rotated among these classrooms. Recruitment began in January 2018. The centre director was first contacted with a recruitment email including eligibility criteria for teachers. Once the centre director agreed to participate, she distributed informed consent forms to interested teachers and all parents. All eligible teachers (i.e. employed full-time at childcare; and tutor children aged 3–5 years) (*n* 16) participated in the study. The total number of eligible children (i.e. typically developing children aged 3–5 years) was 100, and seventy parents returned signed consent forms. All teachers and parents of children who participated in the current study signed and returned written consents. Children provided verbal assent during the data collection.

### Description of the ‘Read for Nutrition’ programme

**Fig. 1:**
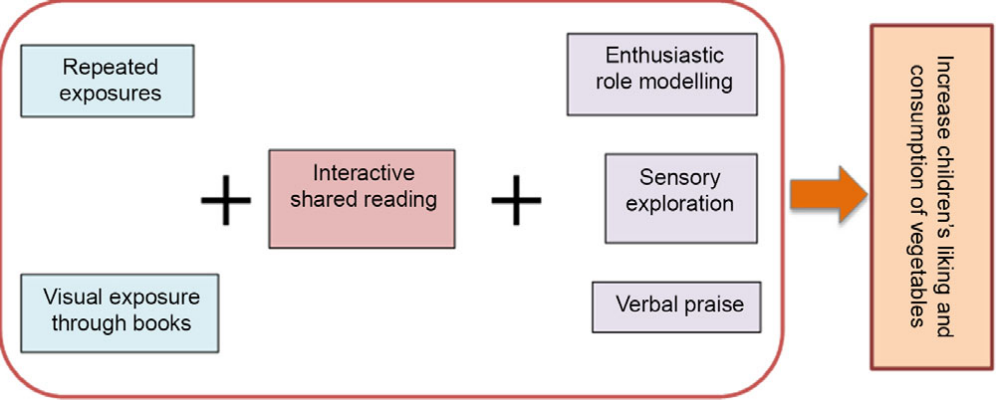
Evidence-based strategies in the ‘Read for Nutrition’ programme

One goal of the ‘Read for Nutrition’ programme is to train teachers on using EBP^(16–18,26)^ during book reading classroom routines (Fig. 1). ‘Read for Nutrition’ is based on the ‘Theory of Mere Exposure’^(34)^ where the development of the target vegetable’s preference and consequent consumption occurs when the child is exposed repeatedly to a positive stimulus.

The ‘Read for Nutrition’ programme consists of a lesson and coaching for ECE teachers and multiple exposures to book reading activity for the children. The lesson is composed of seven topics including introduction and advantages of using books; EBP to use when reading books^(16–18,26)^ to the children; considerations for selecting books; suggestions to overcome barriers; accessing books for nutrition education; activities for practice; additional resources and goal sheet to be used for goal settings during the coaching sessions. The ‘Read for Nutrition’ lesson is included as a supplementary material (S1 Appendix) for review. The programme has been peer-reviewed by a team of five multidisciplinary experts in early childhood/child development, community nutrition and Extension coaches. Further, cognitive interviews with four ECE teachers were conducted prior to implementation. Based on the feedback, programme materials were revised to include more examples of verbal prompts and flash cards to be used during the reading.

After receiving signed consent forms from parents and teachers, baseline data regarding children’s preferences and consumption for broccoli were collected (week 0). Once baseline data collection were completed, each teacher received a copy of the ‘Read for Nutrition’ lesson to read (week 1). Then they received coaching for two weeks (week 2 and 3). Then they received coaching for two weeks using a diagnostic or prescriptive coaching model that focused on helping teachers apply the EBP^(35)^. Coaching included two, one-on-one on-site 45-min sessions with the first author, facilitating reflection, goal-setting and sharing two videos regarding the importance of using the book reading EBP as well as suggestions to overcome barriers^(21)^. Further, the coach provided a reading example with verbal prompts for the book ‘Monsters Don’t Eat Broccoli’^(36)^ for teachers to use as a guide while reading the book.

After two weeks of coaching, the teachers were asked to read the same book ‘Monsters Don’t Eat Broccoli’ multiple times (at least three times/child/week) during the programme (for a total of nine times over a period of 3 weeks). This was done to ensure that the children had been exposed repeatedly to the book before the post-test, as previous research has shown that around five exposures are needed for book reading to be effective with preschool children for behavior change^(26)^. Teachers read the book to a small group of children during circle time. Different classroom teachers would read to a different small group of children each time, and the teacher kept a log sheet to track which children attended each session. After three weeks of intervention, children’s preferences and consumption of broccoli again were collected (week 4). All book reading was in the English language. Each teacher received three in-service hours (programme approved by Nebraska Department of Education) for implementing the programme and a $15 gift card after completion.

### Measures

Children’s demographic information regarding, age, sex and English language proficiency were collected from the centre director. Children’s consumption of broccoli was measured by Food Selection Task^(37)^. In this task, each child was assessed individually by a trained researcher in a separate room in the ECE setting. The target vegetable (broccoli) was served raw, cut to bite size, without any seasoning and cold. Two plates of pre-weighed snacks (0·5 cup/1·5 oz broccoli and 0·5 cup/0·5 oz of cereal as a control food) were placed in front of the child in a standardised format (broccoli on the right and cereal on the left) on separate identical white plates. The same cereal (Multi Grain Cheerios; General Mills, Minneapolis, MN) was used throughout the study. The child was instructed to eat as much or as little as they wish for 10 min. The trained researcher weighed each plate after 10 min using a portable scale (post-weight). The consumption was calculated by subtracting post-weight from the pre-weight to the nearest 0·1 ounces. Children’s proportional product consumption was measured by dividing the weight of each food eaten by the total weight of food eaten. Both total (absolute) and proportional intakes were analysed to understand total amount of foods consumed, because proportional consumption controls for children’s hunger and consider the impact of the picture book is greater for broccoli consumption than for cereal. For example, the impact of the programme will be greater when a child eats 1 ounce target vegetable from 2 ounces of all the foods consumed than 1 ounce target vegetable from 5 ounces of all food consumed^(38)^. This assessment was done at the same time during mid-morning for all the children at both pre- and post-intervention assessment.

Children’s liking of broccoli was measured by using an adapted version of the validated Preschooler Food Liking Assessment Tool^(38–40)^. After measuring the child’s vegetable consumption, the researcher showed the child a three-point rating scale featuring faces of varying levels of enjoyment, i.e. yummy, just okay and yucky to assess the child’s liking of broccoli. Link for the detailed protocol for the measure is included in the S2 Appendix for review.

Acceptability and practicality were evaluated using a self-report survey completed by teachers after receiving and implementing the ‘Read for Nutrition’ programme. Survey questions were adapted from a previous study evaluating a gardening intervention^(41)^. The survey included eight ‘yes/no’ response questions regarding enjoyment of programme strategies; whether these strategies were easy to implement and integrate into existing curriculum and teachers’ perceived effectiveness of the strategies. In addition, teachers were asked to rate implementation of each strategy in their classroom using a three-point Likert scale (easy, sometimes hard and hard).

### Statistical analysis

ANOVA was used to identify baseline differences for proportional consumption (*F* = 1·7, *P* = 0·2) and total consumption (*F* = 0·72, *P* = 0·6) of broccoli. Logistic regression was used to identify baseline differences for liking of broccoli (*R* ^*2*^ = 0·05, *P* = 0·9) during pre-test between children from the five different classrooms. No baseline differences were found between classrooms. Additionally, we have proceeded without including clustering because there was little evidence of children within the same classroom being more similar than children across five classrooms for broccoli consumption prior to the intervention (intracluster correlation coefficient values for pre-consumption of broccoli is, *ρ* = 0 and for pre-proportional consumption of broccoli is, *ρ* = 0·005; *P* = 0·556)^(42)^. Paired sample *t* tests were used to examine the differences in the total and proportional consumption of broccoli between pre- and post-tests. Multiple linear regression analysis was conducted to examine the relationship between post-intervention proportional consumption of broccoli as dependent variable and number of exposures to the book as predictor, controlling for pre-intervention proportional consumption of broccoli, children’s classrooms, children’s age, sex and English language proficiency. Assumptions for normality, homoscedasticity, linearity and multicollinearity were checked before analysis. Wilcoxon signed rank test was used to identify changes in pre- and post-intervention liking for broccoli. Descriptive analysis including means (sd) and frequency distributions was used for the acceptability and practicality survey. All statistical analyses were conducted using SPSS version 25^(43)^. The significance level alpha (*α*) was set at 0·05.

## Results

A total of seventy children and sixteen ECE teachers participated in the study. Children’s average age was 4·26 (sd = 0·68) years. More than half (56·5 %) of the children were female. Nearly half of the children were Non-Hispanic White (49·3 %) and one-third (31·9 %) were Non-Hispanic Black. English was the language spoken at home for 52·9 %. Arabic and Spanish were the languages spoken at home for more than third of the children (21·4 % and 17·1 % respectively). One child left the centre, so was excluded from the analysis; in total, sixty-nine children completed both pre- and post-intervention assessments. All teachers were female and on average were 31 years old (sd = 6·99). Most teachers self-identified as White or Caucasian (75 %) and 13 % as Hispanic or Latino. Over two-thirds (67 %) of teachers had a bachelor’s degree with other teachers having an associate’s degree or attending some college. Teachers had an average of 8·3 (sd = 6·18; range 0·33–19) years of experience at any ECE setting.

Average total consumption of broccoli increased 35 % (0·14 ounces or 0·05th cup) after the ‘Read for Nutrition’ programme (*t* = 2·66; *P* = 0·01; 95 % CIs (0·035, 0·246)) for all children. In addition to increasing the consumption of broccoli, children’s (*n* 69) liking for broccoli (*W* = 2·2; *P* = 0·03; 95 % CIs (0·029, 0·551)) improved after the programme (Table 1). The average proportional consumption of broccoli increased 18·8 % from pre to post intervention; however, the change was NS for all children (*t* = 1·94; *P* = 0·057; 95 % CIs (–0·156, 0·0002)). Mean proportional consumption of broccoli increased by 28 % (0·11 ounces or 0·04th cup) in children (*n* 47) who had received ≥ five exposures to the book reading (*t* = 2·77; *P* = 0·008; 95 % CIs (0·032, 0·20)) (Table 1) with small effect size (Cohen’s *d* = 0·4). The number of exposures to the book was a significant predictor for the post-intervention proportional consumption of broccoli (*β* = 0·365; *P* = 0·002; 95 % CIs (0·026, 0·109)) while controlling for other covariates (Table 2). There were no baseline and post-intervention differences between classrooms for proportional consumption of broccoli across five classrooms. The proportion of children who rated broccoli as ‘Yummy’ increased from 44 % to 61 %. Concomitantly, the proportion of children’s rating for broccoli as ‘Just OK’ and ‘Yucky’ decreased by 5·8 % and 11·6 %, respectively, after the programme.

**Table 1:** Comparison of children’s (*n* 69) total and proportional consumption and liking of broccoli

Measure	Pre		Post		Test of differences	*P*-value
	Mean	sd	Mean	sd		
Proportional consumption of broccoli‡ (oz.)	0·41	0·33	0·49	0·35	1·94†	0·057
Proportional consumption of broccoli (oz.) for children who had 5 or more exposures (*n* 47)	0·39	0·3	0·5	0·35	2·77†	**0·008** **
Total consumption of broccoli (oz.)	0·4	0·42	0·54	0·51	2·66†	**0·01** **
Total consumption of broccoli (oz.) for children who had ≥ 5 exposures (*n* 47)	0·41	0·42	0·6	0·53	2·99†	**0·004** **
Liking of broccoli‖	*n*	%	*n*	%		
Yummy	30	43·5	42	60·9	2·22§	**0·03** *
Just OK	14	20·3	10	14·5		
Yucky	25	36·2	17	24·6		

Note: Boldface indicates statistical significance.

* *P* < 0·05;

** *P* < 0·01;

† Paired-sample *t* test.

‡ Proportional product consumption was measured by dividing the weight of each food eaten by the total weight of food eaten.

§ Wilcoxon signed rank test.

‖ Liking of broccoli was measured using Preschooler Food Liking Assessment Tool^(38–40)^.

**Table 2:** Association between number of exposures to the book ‘Monsters don’t eat Broccoli’ and post-intervention proportional consumption of broccoli†

Dependent Variable	Predictors	Unstandardized coefficients‡	Standardized coefficients‡	*R* ^*2*^-statistic‡	*P*-value‡	Collinearity Tolerance‡	VIF‡
Post-intervention proportional consumption of broccoli§	Number of exposures to the book	0·07	0·4	0·41	**0·002** **	0·81	1·23
	Pre-proportional consumption for broccoli	0·6	0·6		**< 0·001** ***	0·90	1·11
	English Language proficiency	0·05	0·1		0·2	0·86	1·16
	Age	–0·02	–0·04		0·7	0·95	1·05
	Sex	–0·01	–0·02		0·9	0·91	1·1
	Classrom	–0·05	–0·2		0·1	0·90	1·16

Note: Boldface indicates statistical significance.

** *P* < 0·01;

*** *P* < 0·001.

† Brief description of the intervention: Teachers read the ‘Read for Nutrition’ lesson and received coaching for applying evidence based practices outside of the mealtime while reading ‘Monsters don’t eat Broccoli’ multiple times with the children during the three week intervention. Children’s liking and consumption of broccoli were measured as the outcomes of the intervention.

‡ Multiple linear regression analysis.

§ Children’s consumption of broccoli was measured by Food Selection Task^(37)^. Children’s proportional product consumption was measured by dividing the weight of each food eaten by the total weight of food eaten.

All sixteen teachers (100 % response rate) completed the acceptability and practicality questionnaire^(39)^. The programme was rated highly acceptable and practical by the ECE teachers as indicated in their responses. For example, all the teachers reported that they enjoyed applying ‘Read for Nutrition’ strategies in their classroom, and they were willing to use these strategies in their existing curriculum. Descriptive analysis of the teachers’ acceptability and practicality questionnaire is reported in Table 3.

**Table 3:** Early care and education (ECE) teachers’(*n* 16) report of acceptability and practicality of the ‘read for nutrition’ programme

Questions (Yes/No)*	Yes					
	*n*	%				
Did you enjoy applying ‘Read for Nutrition’ strategies in your classroom?	16	100				
Did the children enjoy it?	15	93·8				
Are you willing to use these strategies in your existing curriculum?	16	100				
Do you think it is easy to implement these strategies in your classroom?	16	100				
Do you feel that ‘Read for Nutrition’ is a good idea for increasing children’s exposure to vegetables?	16	100				
Do you think that strategies at ‘Read for Nutrition’ are effective for improving children’s liking/preference of vegetables?	16	100				
Do you think that strategies at ‘Read for Nutrition’ are effective for encouraging children to try/eat vegetables?	16	100				
Were the children in your classroom engaged in story time when you use strategies from ‘Read for Nutrition’?	15	93·8				

Strategies	Easy		Sometimes hard		Hard	
	*n*	%	*n*	%	*n*	%
Using PEER sequence	16	100	0		0	
Using Different Prompts (CROWD)						
a. Completion prompts.	10	62·5	6	37·5	0	
b. Recall prompts.	13	81·3	3	18·8	0	
c. Open ended Prompts.	13	81·3	3	18·8	0	
d. Wh Prompts.	12	75	4	25	0	
e. Distancing prompts.	9	56·3	6	37·5	1	6·3
Engaging senses by asking questions about the taste, color, smell, sound and texture of the vegetables	13	81·3	2	12·5	1	6·3
Role modeling by expressing your liking to the vegetable	15	93·8	1	6·3	0	
Peer modeling by encouraging children to describe their experience	11	68·8	5	31·3	0	
Verbal Praise by praising a child when s/he express liking	16	100	0		0	
Read with enthusiasm	15	93·8	1	6·3	0	

* This survey^39^ has been administered to participating ECE teachers after the programme.

## Discussion

The purposes of the current study were to evaluate if after implementation of ‘Read for Nutrition’ programme, children’s liking and consumption of the target vegetable (broccoli) increased along with testing the acceptability and practicality of the programme in ECE settings. Results from the current study show that the programme has the potential to increase children’s liking and consumption of broccoli and is acceptable for teachers and practical to implement with children.

In line with the hypotheses, after the ‘Read for Nutrition’ programme, the overall consumption and liking of the target vegetable (broccoli) increased significantly for all children. One of the important study findings is that the proportional consumption of broccoli significantly increased only in children who received ≥ five exposures to the book reading experience. This result also identified ‘five times’ as being the threshold level of the exposure, to significantly increase the level of proportional consumption, confirmed by the regression analysis. This finding is supported by previous research showing the numbers of book reading needed in improving children’s consumption of vegetables^(27)^. Future pilot studies should report proportional consumption in addition to total consumption because proportional consumption considers the consumption of the reference food and is therefore more accurate than total consumption of the target food alone^(38)^.

The improvement in consumption and liking for broccoli in the current study are consistent with experimental studies conducted at home^(27)^ and ECE settings^(25,26)^ that showed improvements in children’s liking and consumption of vegetables after book exposure or interactive shared reading congruent with exposure to target vegetables from five days to two weeks^(25–27)^. Regarding the magnitude of the changes, our findings of 0·04th cup increase in proportional consumption and 0·05th cup increase in total consumption of broccoli after the intervention are consistent with previously published meta-analyses reporting an average of 0·12 ounces (0·04th cup) increase in vegetable consumption^(44)^. Vegetables as a food group prevent major public health problems, such as overweight and obesity, cancer and CVD^(45)^, and 93 % children do not meet minimum dietary recommendations for vegetables in the USA^(46)^. Consequently, based on the present study findings, an 18·8 % increase of vegetable consumption in one meal may lead to a total of 56 % increase in vegetable consumption per day, assuming vegetables were served at least three times in a full day ECE. Results from ‘Read for Nutrition’ intervention demonstrate an increase in broccoli consumption that has the potential for increasing children’s preferences for vegetables and hence increasing the chance of meeting children’s dietary recommendations^(2)^ and chronic disease prevention^(45)^.

However, the innovation for the current study is that the exposure to the target vegetable is only through book readings, and taste exposures with real vegetables did not take place as in previous related intervention studies^(25–27)^. Additionally, 17 % more children reported higher liking for broccoli and 11·6 % more children reported lower disliking for broccoli after the programme comparing to children’s pre-test ratings of liking for broccoli. Given the use of a bitter tasting vegetable (broccoli)^(29,43)^, these results are encouraging and consistent with other study finding showing that interactive shared reading increased preschool children’s liking of the target vegetable ‘carrot’ over five consecutive days of intervention^(26)^. Whether continuation of this programme results in increased children’s consumption of the target vegetable during regular mealtime merits further investigation.

An important innovation in the current study is utilising enthusiastic role modelling beyond mealtime into story time. In the ‘Read for Nutrition’ lesson, interactive shared reading was integrated with enthusiastic role modelling^(16)^ by verbal indication of liking (e.g. Yummy, Mmmm) and verbal praise to act as positive reinforcement^(17,18)^. Previous studies reporting that verbal enthusiasm modelling increased consumption more than silent modelling only examined the impact during mealtimes^(16,20)^. The present study findings suggest that story time is an opportunity to teach children about healthy eating and use EBP to positively influence children’s healthy eating. Moreover, the implementation of the ‘Read for Nutrition’ programme requires only a children’s book and low intensity coaching instructions for the teacher. Given that interactive shared reading is a useful strategy for young children to learn vocabulary, develop language^(47,48)^ and increase liking and consumption of vegetables^(26,27)^, ECE stakeholders could encourage teachers to implement this programme in practice.

The ‘Read for Nutrition’ programme was perceived as acceptable and highly practical by ECE teachers and was reported as enjoyable by both children and teachers. Further, the programme addressed previously reported challenges so that nutrition education was easily integrated to the existing ECE classroom book reading routine. Teachers reported that it was mostly easy to use the verbal prompts reflecting the EBP during book reading. All participating teachers indicated that the ‘Read for Nutrition’ strategies were effective in improving children’s liking and consumption of broccoli, addressing previous concerns regarding additional resources and planning when using of food during non-food activities^(21)^.

Future research conducting randomised control trials, with larger sample sizes and with multiple data points from different ECE settings contexts (e.g. family childcare homes and non- Child and Adult Care Food Programme centres), is required to establish causal links between the programme intervention and child-level outcome. In addition, studies are needed to examine the feasibility and effectiveness of these strategies to encourage children’s vegetable consumption during other routine classroom activities, such as circle time. Additionally, the current study could be replicated using a wide range of books describing familiar and unfamiliar vegetables and novel foods, as well as with children who are picky eaters or have food neophobia^(49)^. Lastly, future studies may explore the effectiveness of the programme during regular mealtimes using plate waste data collection methodology. For enhanced scalability and reach for the programme (e.g. to rural or at-risk communities), online professional development coaching through web-based modules or webinars might be an effective option for this promising intervention and worthy of further investigation^(50,51)^.

Limitations of the current study include not having a comparison group, not accounting for presence of siblings in data analysis, not measuring children’s initial familiarity with broccoli and no inclusion of control vegetables. As the initial acceptability of the ‘Read for Nutrition’ programme, a pre-post design was selected. Further, the study included one target vegetable (broccoli) and all participating teachers in the current study were highly educated, worked at one licensed and Child and Adult Care Food Programme-funded centre-based ECE setting, limiting the generalisability of the findings. Lastly, lack of follow-up data collection prevents us from evaluating the programmes long-term impact.

The current study has several strengths. The ‘Read for Nutrition’ lesson includes recommendations for using different children’s vegetable story books, and, therefore, it can be evaluated in future studies and implemented by teachers with a variety of vegetables. Other strengths for the current study include that the liking and consumption of broccoli were measured at the end of the study (delayed affective response) and compared with pre-intervention measures. Thus, the finding was more accurate reflection of liking compared to measuring right after the reading sessions (immediate affective response). Additionally, the current study measured broccoli consumption outside of the regular mealtime that helped avoid confounders including lack of hunger and peers’ and teachers’ influences. Further, proportional consumption of broccoli was considered as the main outcome as it includes consumption of the target food (broccoli) relative to the reference food (cereal). Lastly, the present programme was focused on increasing the liking and consumption of the already familiar but disliked vegetable instead of introducing novel vegetables where change is harder to achieve in this age group^(52)^.

## Conclusion

It is important to educate teachers about practical EBP that can be used in classrooms to improve children’s liking and consumption of vegetables. The present study provides evidence that expands on previous experimental studies evaluating EBP on preschool children’s vegetable intake which can be implemented during classroom routines beyond the mealtime setting.

## Supplementary material

For supplementary material accompanying this paper visit https://doi.org/10.1017/S1368980021004985. click here to view supplementary material
