# Supplementary material for: ‘Read for Nutrition’ programme improves preschool children’s liking and consumption of target vegetable
Source: Public Health Nutr. 2021 Dec 27;25(5):1346–54. doi: 10.1017/S1368980021004985 (PMC9991685; doi:10.1017/S1368980021004985)
Supplement: Supplementary file 1 [file S1368980021004985sup.zip › S1368980021004985sup002.pdf]

READ FOR NUTRITION

Early Childhood Professional Development Program

Authors Names have been removed for the purpose of Blinded Review

Read for Nutrition

Table of Contents

Introduction 2

Advantages of using books 2

Strategies to improve children's preference and consumption of vegetables 3

1. **Interactive shared reading** 3
2. **Role modeling** 4
3. **Modeling by peers or other students** 4
4. **Engage children in food-based sensory exploration** 4
5. **Offer verbal praise to reinforce positive behavior** 4
6. **Be enthusiastic** 4

Putting it All Together: Integrating the Strategies 5

Important Considerations for Choosing Books 6

Barrier to using books in nutrition education and suggestions to overcome this barrier 8

Additional Resources 9

References 10

Activity 11

Goals for the Week Sheet: Read for Nutrition 13

Introduction

Reading books to children is important for improving children's early literacy skills, language development, social-emotional development and math skills. Books are also a great way to teach children about nutrition and where food comes from, and improve their eating habits.

Advantages of using books (https://mediahub.unl.edu/media/4257)

1. **Encourages children to try new vegetables**. Encouraging children to try new vegetables can be challenging. Common strategies such as pressuring or rewarding children to eat can lead to increased fussy eating habits in children.[1] Picture books are an alternative, easy and effective strategy to introduce and encourage children to eat vegetables.[2] Interaction with pictures and stories about new vegetables can promote positive attitudes about the vegetables before the child tastes them.[3]

2. **Increases children's preference and consumption of vegetables.** Some children may not like certain vegetables. However, these foods are important for a healthy diet. When children are exposed to vegetables through books, they may be more willing to like and eat vegetables.[5]

Reading a book to children about vegetables in not enough. It is important to follow specific strategies while reading to children to improve their vegetable liking and consumption.

Throughout this lesson you will learn how to use books to improve children's vegetable preference and consumption.

Teacher Titbit

When children are exposed to vegetables through picture books in an engaging and interactive way, it will increase their familiarity, willingness to try and liking of the vegetable.

Strategies to improve children's preference and consumption of vegetables

1. **Interactive shared reading**

   During interactive shared reading, the childcare provider **actively engages the child** while reading to the child.

   The childcare provider can implement interactive shared reading through a technique called the **PEER** sequence.[6]

   Use the PEER sequence while reading with the children.

**P**rompt the child to say something about the book	*"Tell me what you see."*
**E**valuate the child's response	*"Yes, that's right."*
**E**xpand the child's response by rephrasing and adding information to it.	*"That's a red, delicious radish. It helps our bodies be healthy."*
**R**epeat the prompt to make sure the child has learned from the expansion	*"Tell me what else is red."*
* If the child is wrong, you can offer paraphrase reflections to clarify children's thinking and help other children make observations or connections, and re-direct when necessary. Such as: *"Maha said this is a radish. It does look like a radish because it's red. It's a tomato. It's rounder and bigger than a radish and has a green stem. Have you ever eaten a tomato?"*	

Use the CROWD sequence to use different prompts while reading with the children. Below are examples of prompts that you can use:

**C**ompletion prompts	You leave a blank at the end of a sentence and get the child to fill it. You typically use these prompts in books with rhyme or books with repetitive phrases; however, you can also use the picture in the books to indicate the word that they could use to complete the statement.<br>For example: *"The monster would rather eat ______________."*
**R**ecall prompts	Ask children to recall information from the story, such as characters, sequence, or plot.<br>For example: *"Can you tell me what happened to the monsters when they went to the restaurant?"*
**O**pen-ended prompts	These prompts focus on the pictures in the books. They work best with books that have rich, detailed illustrations.<br>For example: While looking at a page, the childcare provider may ask, *"Tell me what's happening in this picture."*
**W**h prompts	These prompts usually begin with what, where, when, why, and how questions that focus on the pictures in the books.<br>For example: *"What is the food that the monsters do not eat?"*
**D**istancing prompts	These prompts ask children to relate the pictures or words in the book they are reading to experiences outside the book.<br>For example: While looking at the book with a picture of broccoli, *"When was the last time we had broccoli for lunch?"*

2. **Role modeling**
   Role modeling is leading by example. Children mimic adults, therefore, adults can show children how to do something and provide children with examples of a desired behavior.

   While reading the book, you can indicate your liking to the vegetable or describe a situation where you tried the vegetable and liked it. For example: *"I love broccoli. This is my favorite vegetable*." If you express your liking for a vegetable, children will be more willing to try it.

3. **Modeling by peers or other students**
   Childcare setting is ideal for using peer modeling, because children are present in a group. Encourage children who liked a vegetable to talk about it during the group reading session.

   For example: The childcare provider says, *"John told me that he tried broccoli yesterday at his grandma's house and it was delicious. Good job, John! Broccoli is very tasty and healthy for you! John, would you like to share with your friends how good the broccoli was for you?"*

4. **Engage children in food-based sensory exploration**
   When children have the opportunity to explore the food with their five senses (sight, smell, touch, hearing and taste) they are more likely to try the food.

   While reading the book, you can ask children questions about the color, smell, sound, texture and taste of the vegetable.

5. **Offer verbal praise to reinforce positive behavior**
   Praise a child when s/he tells about their experience trying a vegetable or expresses liking a vegetable.

   For example: While reading a book about carrots, one child said *"I ate a carrot yesterday."*
   So the childcare provider said *"Good job eating carrots. They are very crunchy. Did you like it?"*

6. **Be enthusiastic**
   Read to children with enthusiasm, different voice tones, and positive language.

Putting it All Together: Integrating the Strategies

Below is a table that describes how the childcare provider integrated the strategies above using examples from the book *Monsters Don't Eat Broccoli*.

Integration of Interactive Shared Reading and Nutrition Strategies	Example
**Be enthusiastic.** The childcare provider is being **enthusiastic.** She shows a picture of the vegetable to the class.<br><br>**Role modeling.** The childcare provider is implementing role modeling as she indicates she likes broccoli.<br><br>**Completion prompt.** The childcare provider used a completion prompt by leaving a blank at the end of a sentence to let the children fill it in.	Childcare provider: "*Good morning children! Are you ready for a story? We will read a book about one of my favorite vegetables.* (Point to the picture of the vegetable shown on the first page of the book) *Who knows what this vegetable is called?* ***Its name is ________. I love it. It is delicious!***"<br><br>Childcare provider: "*Ok, now I have a surprise for you. We will have a new vegetable in our lunch today. We will eat broccoli!"*
**Be enthusiastic.** The childcare provider is being **enthusiastic.** She is using positive language as she introduces the book to the children.<br><br>**Wh prompts and open-ended prompts.** The childcare provider used the wh prompt by asking the children 'where' and the open-ended prompt by asking the children about the picture in the book.	Childcare provider: *"I would like to share* ***this awesome book*** *called* Monsters Don't Eat Broccoli. *Maybe this book will help us know more about broccoli before trying it!* ***Where is the broccoli*** *on this page?* ***Tell me what's happening in this picture.***"
**Recall prompts.** The childcare provider used the recall prompt by asking children to recall information from the story.	While reading, "*Monsters did not want to eat broccoli at first, but then they changed their mind.* ***Tell me why they changed their mind."***
**Sensory exploration.** The childcare provider is promoting the sense of hearing.<br><br>**Enthusiastic role modeling.** She is modeling and being enthusiastic in order to encourage the children to try the broccoli.<br><br>**Distancing prompt.** The childcare provider is using distancing prompts in order to connect the book and the past/future experience.	Childcare provider: "*Wow,* ***I love broccoli****. This vegetable is* ***delicious****. It is* ***crunchy when it's raw. Have you listened to the crunchy sound****?*" (Trying to imitate the crunchy sound.)

Important Considerations for Choosing Books

I. First, it is important to consider the characteristics of the book, such as:

1. **Entertainment Education (EE).** Entertainment education simply means to deliver educational messages through fun and entertaining narratives.

   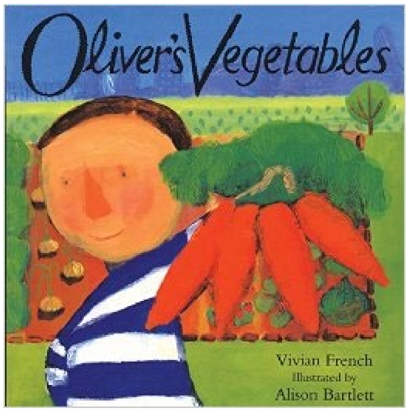

   EE has two defining characteristics:[5]

   A) EE-productions contain an ***educational*** message. For example, eating carrots makes you feel fit and strong.

   B) The educational message is incorporated into an ***entertaining*** narrative, such as a story about the adventures of a rabbit.

   Oliver's Vegetables provides an educational message, which is teaching kids about different kinds of vegetables. It also incorporates an entertaining narrative, which is Oliver's trip to his grandpa's garden.

2. **Colorful, realistic pictures.** Transfer of learning is best supported by pictures that closely resemble their real-world referents; thus, realistic photographic images facilitate generalization of learning compared to less realistic images.[5]

   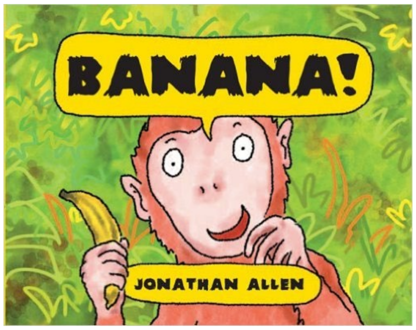

   For example, the book *Growing Colors* is full of gorgeous photographs of 14 fruits and vegetables of different colors.

3. **Character–product congruence.** Children like fruits and vegetables when combined with congruent characters that are associated with those fruits or vegetables—like monkeys with bananas and rabbits with carrots.[5]

   The book *Banana!* has a story of a monkey to promote eating of bananas. In addition, the book *Carrot Soup* features the story of a rabbit for promoting carrots.

II. Second, identify the type of book to use according to your target age group, such as:

- <u>Younger children (2-3 years old)</u>. Picture books are good to promote visual exposure and increase familiarity to fruits and vegetables. It is important to expose children to as many pictures of healthy food as you can to increase their willingness to try them. These kinds of books work best with younger children.
- <u>Older children (4-5 years old)</u>. Story books teach nutrition principles, like the importance of eating a variety of food and how to make healthy food choices. Story books combine the nutrition message in an entertaining narrative (story) to make it easier for the young children, with developing cognitive abilities, to understand.

Let's look at a list of books based on the mentioned characteristics.

**Name of the Book**	**Why is this book a good choice?**	
1. *I Will Never Not Ever Eat a Tomato* http://www.amazon.com/Will-Never-Ever-Tomato-Charlie/dp/0763621803	This story book has an entertaining narrative which encourages kids to try new fruits and vegetables. Also, the pictures are realistic photographic pictures.<br>In this story, Charlie tricks his sister into trying new foods by calling them catchable, interesting names like *orange twiglets from Jupiter* for carrots and *green drops* for peas.<br>This book is suitable for children 3+ years.	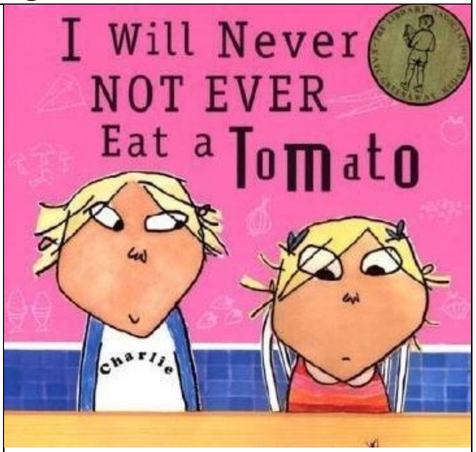
2. *An Alphabet Salad: Fruits and Vegetables from A to Z* http://www.amazon.com/Alphabet-Salad-Fruits-Vegetables-Books/dp/0736816836	This picture book consists of large, colorful photographs of a fruit or vegetable for each letter of the alphabet.	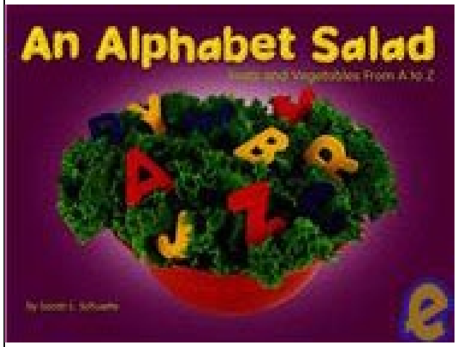
3. *Rah, Rah, Radishes! A Vegetable Chant* http://www.amazon.com/Rah-Radishes-Vegetable-Chant-Classic/dp/1442499273	This picture book features chants for children regarding fresh vegetables. It also includes photographs of vegetables in a variety of settings, like farmer's markets, grocery stores, and more.<br>This book is suitable for children 2-5 years.	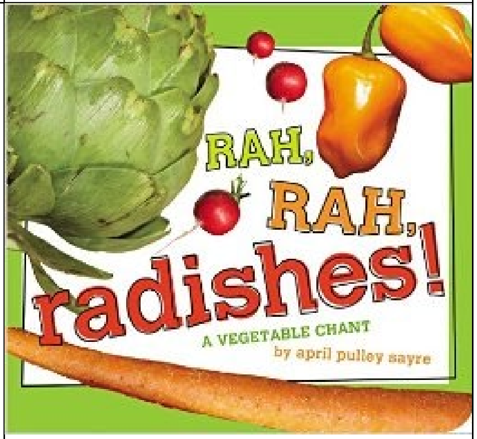

4. *Cool as a Cucumber*	This picture book is a sturdy, fold-out concept book that introduces toddlers to eight common vegetables using colorful, appealing photographs with minimal text. Each four-page section begins with a raw vegetable and a photograph of it before it is harvested. Next, it shows a young child getting ready to eat the vegetable and the various ways it is prepared. The section concludes with comparing an attribute of the vegetable to several other kinds of food.	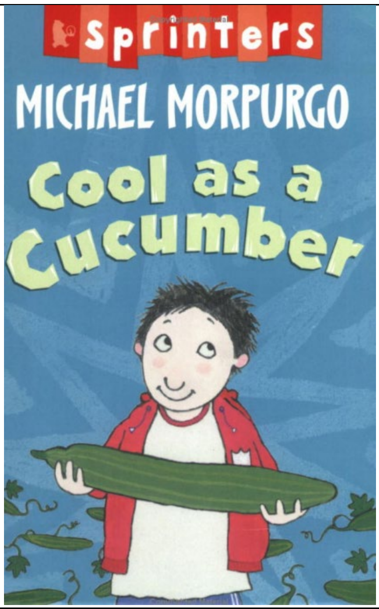

Barrier to using books in nutrition education and suggestions to overcome this barrier
Barrier. It is hard for me to read books to children, because we don't have the resources to buy books.

Lacking resources? Watch this video: http://mediahub.unl.edu/media/4256.

Resources to find books:

- **Public library**. A local public library can be a great place to get access to books. You can also ask local book mobiles to use your center as a community location and let kids enjoy a mini field trip.
    - Here is the link to Lincoln public libraries website: http://lincolnlibraries.org/

> **Teacher Titbit**
>
> *You can hold the books you need through the library website. This way you will find them waiting for you at your preferred library branch!*

- **Reach out to Nebraska Extension.** The goal of Nebraska Extension is to provide resources to the public. Reach out to any of the individuals in Nebraska Extension to access free resources: http://childpd.unl.edu/index.php?menu=about
- **Order free materials from Team Nutrition.** This a great way to get free materials. The Team Nutrition website: https://pueblo.gpo.gov/FNS/FNSPubs.php.
- **First book website.** *First book* offers high quality books at deep discounts to schools and programs serving children in need: http://www.firstbook.org/receive-books

Additional Resources

1. IGrow readers curriculum: Developed by South Dakota State University. This program pairs children's books such as *Little Red Hen*, *Bread & Jam for Francis,* and *Grandmother Spider Brings the Sun*, with activities that promote healthy eating and physical activity.
http://igrow.org/healthy-families/health-and-wellness/igrow-readers/

2. Texas Department of State Health Services: Nutrition book suggestions and lesson plans
http://www.dshs.state.tx.us/kids/lessonplans/

3. Florida Department of Health: Children books
http://www.floridahealth.gov/programs-and-services/childrens-health/child-care-food-program/nutrition/preschool-lesson-plans.html

4. Team Nutrition *My Plate* e-books
http://www.fns.usda.gov/tn/myplate-ebooks

5. Using Children's Storybooks as a Basis for Nutrition Education – Webinar
http://www.nfsmi.org/ResourceOverview.aspx?ID=282

6. Super Kids Nutrition Website: Children's book corner
http://www.superkidsnutrition.com/nutrition_resources/bookcorner/

7. Healthy Habits for Life Resource Kit: Download the bilingual storybook to share with your class.
http://classroom.kidshealth.org/index.jsp?Grade=cc&Section=hhfl

8. Online story "My Vegetable Friends"
http://storyberries.com/my-vegetable-friends/

9. Nutrition education resources for preschool
http://healthymeals.nal.usda.gov/nutrition-education/nutrition-education-resources-preschool

10. Reading rockets website: resources for preschool teachers and childcare providers
http://www.readingrockets.org/audience/professionals/preschool

References:

1. Byrne E, Nitzke S. Preschool children's acceptance of a novel vegetable following exposure to messages in a storybook. *Journal of Nutrition Education and Behavior*. 2002;34(4):211-214.

2. Johnson SL. Developmental and environmental influences on young children's vegetable preferences and consumption. *Adv Nutr*. 2016;7(1):220S-231S. doi: 10.3945/an.115.008706 [doi].

3. Houston-Price C, Burton E, Hickinson R, et al. Picture book exposure elicits positive visual preferences in toddlers. *J Exp Child Psychol*. 2009;104(1):89-104.

4. Heath P, Houston-Price C, Kennedy OB. Increasing food familiarity without the tears. A role for visual exposure? *Appetite*. 2011;57(3):832-838.

5. de Droog SM, Buijzen M, Valkenburg PM. Enhancing children's vegetable consumption using vegetable-promoting picture books. The impact of interactive shared reading and character–product congruence. *Appetite*. 2014;73:73-80.

6. Whitehurst GJ, Arnold DS, Epstein JN, Angell AL, Smith M, Fischel JE. A picture book, reading intervention in day care and home, for children from low-income families. *Dev Psychol*. 1994;30(5):679.

7. Dazeley P, Houston-Price C. Exposure to foods' non-taste sensory properties. A nursery intervention to increase children's willingness to try fruits and vegetables. *Appetite*. 2015;84:1-6.

8. Hendy HM. Comparison of five teacher actions to encourage children's new food acceptance. *Annals of Behavioral Medicine*. 1999;21(1):20-26.

9. Cooke LJ, Chambers LC, Añez EV, Wardle J. Facilitating or undermining? The effect of reward on food acceptance. A narrative review. *Appetite*. 2011;57(2):493-497.

Activity

It is time to practice! Let's integrate prompts of interactive shared reading and strategies to promote nutrition education for young children in the classroom. How could you deal with the following situations? Using the following pages as inspiration, which activities would you create using prompts of interactive shared reading and nutrition strategies to promote children's preference and consumption of vegetables for children?

Page	Completion prompts	Recall prompts	Open-ended prompts	Wh prompts	Distancing prompts	Sensory exploration of food	Modeling healthy eating behaviors	Enthusiasm
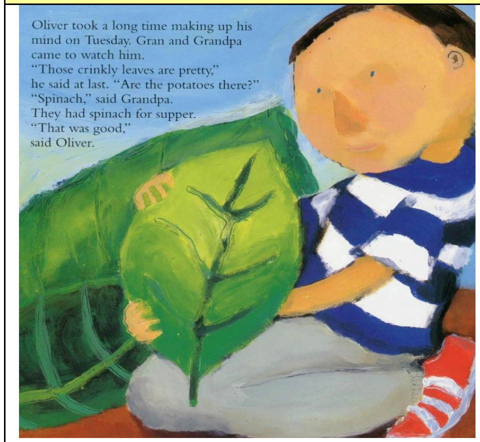		Tell me the name of the new vegetable that Oliver discovers.				Is the spinach leaf smooth and flat?	I love spinach! It is very juicy!	

Page	Completion prompts	Recall prompts	Open-ended prompts	Wh prompts	Distancing prompts	Sensory exploration of food	Modeling healthy eating behaviors	Enthusiasm
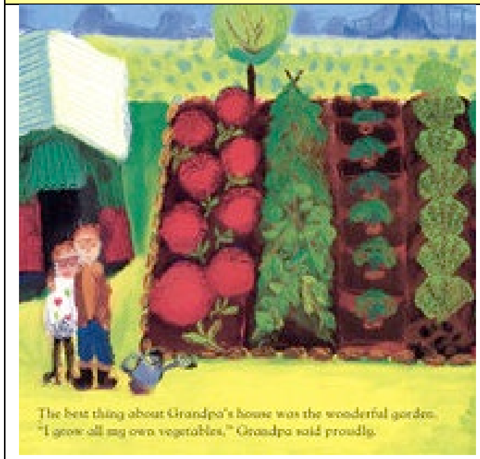 	The grandpa will grow his own_____.		Tell me what's happening in this picture.		Do you remember when we touched the spinach?<br><br>Was the leaf smooth like paper?<br><br>Or was the leaf crinkly like this bag?			

Goals for the Week Sheet: Read for Nutrition

Now that you have completed the Read for Nutrition Lesson, let's think about what you may still have questions about. In terms of role modeling during mealtimes…

I understand ____.

A confusing part was ____.

I'm still wondering ____.

I think I'll understand why or how to use these strategies better if ____________________________________.

Instructions:

Goal setting is an important part of implementing what you have learned and overcoming possible challenges. Below are the strategies discussed in this training. As you are working on these strategies, think about them as learning goals. **Rate where you are in your learning by indicating your current practice for each strategy. Think about possible challenges and how to overcome any challenges that may come up when trying to use the strategies.**

**Goal**	**Current practice**			**Possible challenges**	**Some ways that you can overcome these challenges**
			**A. Promoting Interactive Reading**		
**1. Using PEER sequence**<br>**P**rompt the child to say something about the book.<br>**E**valuate the child's response.<br>**E**xpand the child's response by rephrasing and adding information to it.<br>**R**epeat the prompt to make sure the child has learned from the expansion.	Not doing this yet.	I do this sometimes, but I need more practice.	I understand the importance of this and I do it!		
**2. Using different prompts (CROWD)**<br>**C**ompletion prompts<br>**R**ecall prompts<br>**O**pen-ended prompts<br>**Wh** prompts<br>**D**istancing prompts	Not doing this yet.	I do this sometimes, but I need more practice.	I understand the importance of this and I do it!		

B. Using Nutrition Strategies					
**Role modeling**<br>Role modeling (e.g., express your liking to the vegetable, telling your experience with the vegetable)	Not doing this yet.	I do this sometimes, but I need more practice.	I understand the importance of this and I do it!		
**Modeling by peers or other students**<br>Peer modeling (e.g., encourage children to describe their experience)	Not doing this yet.	I do this sometimes, but I need more practice.	I understand the importance of this and I do it!		
**Engaging children's senses**<br>Asking questions about the taste, color, smell, sound and texture of the vegetables	Not doing this yet.	I do this sometimes, but I need more practice.	I understand the importance of this and I do it!		
**Using verbal praise**<br>Praise a child when s/he expresses liking	Not doing this yet.	I do this sometimes, but I need more practice.	I understand the importance of this and I do it!		
**Being enthusiastic**<br>Reading with enthusiasm	Not doing this yet.	I do this sometimes, but I need more practice.	I understand the importance of this and I do it!		
**Being positive**<br>Using positive language	Not doing this yet.	I do this sometimes, but I need more practice.	I understand the importance of this and I do it!		
