# Supplementary material for: ‘Read for Nutrition’ programme improves preschool children’s liking and consumption of target vegetable
Source: Public Health Nutr. 2021 Dec 27;25(5):1346–54. doi: 10.1017/S1368980021004985 (PMC9991685; doi:10.1017/S1368980021004985)
Supplement: Supplementary file 1 [file S1368980021004985sup.zip › S1368980021004985sup003.docx]

**S2 Appendix: Protocol for children’s preference and consumption of Broccoli ^35-38^**

**Description**

Each child will be assessed individually by a trained evaluator in a separate room in the childcare center. Target vegetable will be prepared without any seasoning, served raw or branched and cold. Two plates of pre-weighed snacks (the target vegetable and a control food) will be placed in front of the child in a standardized format (target vegetable on the right and dry unsweetened cereal on the left) on separate identical white plates. The child will be instructed to eat as much or as little as they wish for 10 minutes.

The trained evaluator will weigh each plate after the child leaves using a transportable scale (post-weight).

After the food selection task, children’s liking of the vegetable will be assessed by asking the children to describe how they like the target vegetable, using a 3-point Likert-like scale featuring faces of varying levels of enjoyment (items labeled yummy, okay, and yucky) using an adapted version of the validated Preschooler Food Preference Assessment Tool.

**Materials Needed**

- 0.5 cup of cheerios for each child
- 0.5 cup of broccoli for each child
- 2 plates for each child
- Gloves
- Small bottle of water
- Yummy, Yucky and Just OK faces
- Scale
- 2 Measurement cups (0.5 cup)
- Cutlery
- Stickers

**Set Up**

Broccoli

Cheerios

Researcher

Child

1. First, wash your hands with soap and warm water thoroughly.

2. Wear a new glove for each child before dealing with any food. Try not to touch the food before the child eats.

3. You will be provided with a container for cheerios and another one for washed broccoli with a standardized measuring cups for each. Please keep each cup for the same food at all times.

**Before the child comes-**

1. Write the child (First and Last) name on the log sheet.
2. Switch the scale on.
3. Select the unit to be oz.
4. Put half a cup of cheerios on a plate.
5. Record the weight of cheerios with the plate at the pre column in the log sheet (write the weight exactly as shown on the scale, don’t round)
6. Put the plate of cheerios on the table (left to the child) as shown in the figure.

Repeat the previous steps for the broccoli.

1. Switch the scale on.
2. Select the unit to be oz.
3. Put half a cup of broccoli.
4. Record the weight of broccoli with the plate at the pre column of broccoli in the log sheet (write the weight exactly as shown on the scale, don’t round)
5. Put the plate of broccoli on the table (right to the child) as shown in the figure.
6. Add a small bottle of water and cutlery to be visible to the child.

**When the child comes-**

1. The researcher tells the child: “Hi! My name is -------- what is your name?” “I have some food for you today, eat as much or as little as you like from each plate for 10 minutes and I will let you know when the time is over” – “water and spoons or forks are available for you if you need”.
2. The researcher and the child sit together at the table as shown in the above figure.
3. The child should be allowed to explore or eat for 10 minutes – set the timer to 10 minutes once the child sit.
4. After the ten minutes are over, tell the child:” Ok now we are done with this part.”
5. Remove the plates from the sight of the child – you will weight them later after the child leaves.
6. Show the child the three faces and explain them to the children as below:

•For the ‘**yummy'** face the child will be told that ``this is the face that you might make when you taste something really nice and delicious; it's a yummy face''.

•For the ‘**yucky’** face; ``this is the face that you might make when you taste something you really don't like; it's a yucky face''.

•For the ‘**just okay’** face the child will be told that ``this is the face that you make when you taste something that doesn't taste really yummy and doesn’t taste really yucky; it just tastes okay''.

It is important to make sure that the child understood the three faces by asking the child: “which of these faces would you use for a Yummy face? Which face would show a Yucky face? Which face would you use to show a just OK face?'' If the child answer correctly move on to the next step, if not repeat this step.

1. Tell the child “Now I want you to tell me what you think about broccoli using these 3 faces.” Record the child response in the log sheet – if the child does not respond, prompt the child by pointing to the three faces and asking the child “which face do you make when you eat broccoli?”
2. Offer the child a sticker.

**After the child leaves-**

1. Return any spills to its plate before weighing.
2. Record the weight of remaining cheerios with the plate at the post column of cheerios in the log sheet.
3. Record the weight of remaining broccoli with the plate at the post column of broccoli in the log sheet.
4. Discard the plates with any remaining food after weighing.

**References and Protocol Adaptation**

35. Staiano AE, Marker AM, Frelier JM, Hsia DS, Martin CK. Influence of Screen-Based Peer Modeling on Preschool Children’s Vegetable Consumption and Preferences. *J Nutr Educ Behav*. 2016;48(5):331-335. e1.

*Protocol Adaptation: Staiano et al. (2016) is the reference for the food selection task. We did the same methods as described in this paper except that their target vegetable was bell peppers and ours was Broccoli

36. Birch LL. Dimensions of preschool children’s food preferences. *J Nutr Educ*. 1979;11(2):77-80.

*Protocol Adaptation: Birch (1979) is the first one who used the faces (the original measure)- In the papers I studied, this is cited as the first one to develop a measure for preference for this age.

37. Izumi BT, Eckhardt CL, Hallman JA, Herro K, Barberis DA. Harvest for healthy kids pilot study: associations between exposure to a farm-to-preschool intervention and willingness to try and liking of target fruits and vegetables among low-income children in head start. *J Acad Nutr Diet*. 2015;115(12):2003-2013.

*Protocol Adaptation: Izumi et al. (2015) used the same preference measure we used except that they used it for multiple target vegetables, the method and the explanation of faces to the children for training are mentioned in this paper

38. Jaramillo SJ, Yang S-J, Hughes SO, Fisher JO, Morales M, Nicklas TA. Interactive computerized fruit and vegetable preference measure for African-American and Hispanic preschoolers. *J Nutr Educ Behav*. 2006;38(6):352-359.

*Protocol Adaptation: Jaramillo et al. (2006) is the same measure for preference. They published a picture of how the faces looks like and we used similar faces to the ones published in this study.

**Log sheet**

Child name: -----------------------------------

Researcher Name: ------------------------

Date: ---------------------------------

**Food Selection task**

| **Pre/post** | **Pre** | | **Post** | |
| --- | --- | --- | --- | --- |
| Food | Cheerios | Broccoli | Cheerios | Broccoli |
| Weight in ounces |  |  |  |  |

**Food Preference for Broccoli**

|  |  | **Check** |
| --- | --- | --- |
| 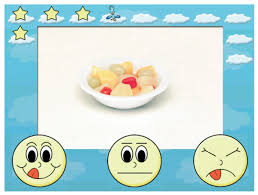 | Yummy  I like it |  |
| 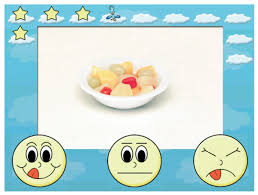 | It’s OK |  |
| 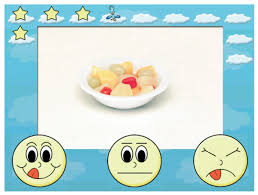 | Yucky  I don’t like it |  |

**Notes: (Write any notes for example if the child was shy, did not seem to understand the faces .. etc)**
